# Emphasizing the Health Benefits of Vitamin D for Those with Neurodevelopmental Disorders and Intellectual Disabilities

**DOI:** 10.3390/nu7031538

**Published:** 2015-02-27

**Authors:** William B. Grant, Sunil J. Wimalawansa, Michael F. Holick, John J. Cannell, Pawel Pludowski, Joan M. Lappe, Mary Pittaway, Philip May

**Affiliations:** 1 Sunlight, Nutrition, and Health Research Center, PO Box 641603, San Francisco, CA 94164-1603, USA; 2 Department of Medicine & Endocrinology, Cardio Metabolic Institute, Somerset, NJ 08873, USA; E-Mail: suniljw@hotmail.com; 3 Department of Medicine, Section of Endocrinology, Nutrition, and Diabetes, and the Vitamin D, Skin, and Bone Research Laboratory, Boston University Medical Center, Boston, MA 02118, USA; E-Mail: mfholick@bu.edu; 4 Vitamin D Council and San Luis Obispo Integrative Medicine, San Luis Obispo, CA 93401, USA; E-Mail: jjcannell@vitamindcouncil.org; 5 Department of Biochemistry, Radioimmunology, and Experimental Medicine, The Children’s Memorial Health Institute, 04-730 Warsaw, Poland; E-Mail: pludowski@yahoo.com; 6 Creighton University School of Medicine, Omaha, NE 68131, USA; E-Mail: joanlappe@creighton.edu; 7 Global Clinical Advisor-Health Promotion, Special Olympics International and Affiliate Faculty, College of Education and Human Sciences, University of Montana, Missoula, MT 59812, USA; E-Mail: mpitt59802@aol.com; 8 International Foundation for Chronic Disabilities, Inc., PO Box 166, Oxford, NJ 07863, USA; E-Mail: pmay1@mindspring.com

**Keywords:** autism, bone health, cancer, cardiovascular disease, developmental disabilities, down syndrome, fractures, intellectual disabilities, vitamin D, 25-hydroxyvitamin D

## Abstract

People with neurodevelopmental disorders and intellectual disabilities have much greater health care needs. Mainly staying indoors, such people generally have low 25-hydroxyvitamin D (25(OH)D) concentrations. The Vitamin D Task Force of the American Academy of Developmental Medicine and Dentistry (AADMD) reviewed the evidence of 25(OH)D concentrations that benefit the health of persons with developmental disabilities. Maintaining recommended optimal serum 25(OH)D concentrations year long will benefit skeletal development in infants, children, and adolescents, and benefit musculoskeletal health and neuromuscular coordination in adult patients, and decrease risk of falls. Maintaining optimal concentrations decreases risks and severities of autoimmune diseases, cardiovascular disease, many types of cancer, dementia, types 1 and 2 diabetes mellitus, and respiratory tract infections. Other benefits include improved dental and oral health and improved physical performance. The Task Force recommends that 25(OH)D concentrations for optimal health to be in the range of 75 to 125 nmol/L, which can be achieved using between 800 and 4000 IU/day vitamin D_3_ and sensible exposure to solar UVB radiation. The paper also discusses the potential risks of higher 25(OH)D concentrations, the evidence from and limitations of randomized controlled trials, and the recommendations by various groups and agencies.

## 1. Introduction

People with neurodevelopmental disorders and intellectual developmental disabilities (IDD), or medically complex developmental disabilities (MCDD), require much greater health care than other patient populations. According to the American Academy of Developmental Medicine and Dentistry (AADMD), the most commonly diagnosed neurodevelopmental disorders are Down syndrome, fetal alcohol spectrum disorder, fragile X syndrome, cerebral palsy, autism, and intellectual disability of unknown origin [1]. The Canadian consensus guidelines for adults with developmental disabilities [2] summarize these and related issues for such people in Canada.

People with MCDD are prone to having low blood concentrations of 25-hydroxyvitamin D (25(OH)D) for several reasons, including generally staying indoors or excessive use of sunscreens, propensity to obesity, and taking various medications [3]. These people therefore have higher rates of osteopenia and osteoporosis [4], chronic diseases, [5,6,7,8], respiratory infections [9], and poorer oral health [10] than community-dwelling individuals. Evidence suggests that vitamin D offers several health benefits, including reduced risk of falls and fractures, several types of cancer, cardiovascular disease, cognitive decline, dementia, diabetes mellitus, respiratory and other types of infections, and many other conditions and diseases [11].

In light of the rapidly advancing understanding of vitamin D’s importance for optimal health, AADMD commissioned its Vitamin D Task Force to review evidence of vitamin D’s health benefits and recommend strategies to manage vitamin D deficiency among the MCDD community. Since those in the MCDD community are living longer now [12], the task force considered the effects of vitamin D for all age groups.

## 2. Approach and Rationale

In carrying out this charter, task force members used PubMed.gov and other databases to review the literature on the health conditions of people with MCDD as well as on vitamin D’s health benefits. Members used the following search terms: vitamin D and intellectual disabilities, developmental disabilities, many diseases; developmental disabilities or intellectual disabilities and health outcomes. Many findings supporting vitamin D’s role in reducing risk of disease come from observational studies that determined health outcomes from studies measuring blood 25(OH)D concentrations at time of enrollment or diagnosis. One can raise 25(OH)D concentrations by either UVB exposure or oral vitamin D intake. Thus, it is possible that 25(OH)D concentrations are, in part, an index of solar UVB exposure that includes effects other than vitamin D production.

Concern has been raised that 25(OH)D concentration-health outcome relations could be due to reverse causation, that is, that having disease affects 25(OH)D concentrations, since few randomized controlled trials (RCTs) have supported the findings of observational studies in general. The reason for this is thought to be largely because of trial design. Most vitamin D RCTs were based on the pharmaceutical drug model and assume that the only source of the agent is through the trial and that a linear dose-response relation exists; neither assumption is valid for vitamin D [13]. In addition, many trials used little vitamin D (400–1000 IU/day), did not measure 25(OH)D concentrations at time of enrollment or end, and enrolled mostly people with 25(OH)D concentrations near or above 50 nmol/L. Thus, one would not expect such studies to find significant benefits. Thus, for now, observational studies—especially in the form of meta-analyses—appear to offer the best information on the link between vitamin D and many health outcomes [11,14].

We intend these guidelines to prevent chronic and infectious diseases, not treat them, and several papers discussed here based their vitamin D guidelines and recommendations largely on observational studies.

## 3. Findings

The first task was to determine what conditions and diseases are more common among those with MCDD. Table 1 shows representative findings for several vitamin D-sensitive diseases. Rates for many chronic diseases are approximately twice those for community-dwelling, non-MCDD individuals. Table 2 gives the definitions of the acronyms regarding disabilities.

**Table 1:** Representative findings regarding disease rates among those with developmental and/or intellectual disabilities.

Disease	Population	Finding	Reference
Cancer	US 2006–2012 NationalHealth Interview Survey	OR = 1.61 (95% CI, 1.34, 1.94)	[8]
Chronic kidney disease	Adults older than 50 years with ID in The Netherlands	Prevalence = 15.3%	[15]
Coronary heart disease	US 2006–2012 National Health Interview Survey	OR = 2.92 (95% CI, 2.33, 3.66)	[8]
Diabetes mellitus	US 2006–2012 NationalHealthInterview Survey	OR = 2.57 (95% CI, 2.10, 3.15)	[8]
Fractures	Adults with Down syndrome or DD, Wisconsin	32% (30/93) of charts contained history of an adult-onset fracture	[16]
Hypertension	US 2006–2012 National Health Interview Survey	OR = 2.18 (95% CI, 1.94, 2.45)	[8]
Obesity	US 2006–2012 National Health Interview Survey	OR = 1.81 (95% CI, 1.63, 2.01)	[8]
Oral health	Adults with IDDs dental care from state-supported dental clinics	Untreated caries, 32.2%; periodontitis, 80.3%; edentulism, 10.9%	[17]
Osteopenia, osteoporosis	Community-dwelling individuals with DD and/or ID in Tennessee	Osteopenia, 51%; osteoporosis of femur bone, 17.1%	[18]
Respiratory infections	6-month-long observational cohort study with 63 persons with IDD	(35% of participants): 12 pneumonias, 7 sinusitis, 1 bronchitis, and 1 upper respiratory tract infection	[19]
	Those with Down syndrome	High prevalence	[20]
Sarcopenia	Adults older than 50 years with ID in The Netherlands	Prevalence = 14.3%	[21]

DD, developmental disabilities; ID, intellectual disability; IDD, intellectual and developmental disabilities; OR, odds ratio.

**Table 2:** Definitions.

DD	Developmental disability (DD) is a diverse group of severe chronic conditions due to mental and/or physical impairments. Developmental disabilities cause individuals living with them many difficulties in certain areas of life, especially in “language, mobility, learning, self-help, and independent living” [22].
ID	Intellectual disability is a disability characterized by significant limitations in both intellectual functioning and adaptive behavior, which covers many everyday social and practical skills. This disability originates before the age of 18 [23].
IDD	Intellectual and developmental disability is a combination of ID and DD.
MCDD	Multiple complex developmental disorder is a category proposed to involve several neurological and psychological symptoms where at least some symptoms are first noticed during early childhood and persist throughout life, including both pervasive developmental disorder and psychosis.

Respiratory tract infections (RTIs) are common among those with developmental disabilities [19,24] because they: (1) often live together in group homes or institutions, where RTIs can spread rapidly; and (2) generally have low serum 25(OH)D concentrations due partly to staying largely indoors. Also, some medicines given to this population, such as anticonvulsant drugs, glucocorticoids, and AIDS medications, reduce serum 25(OH)D concentrations [25].

Similarly, the MCDD community has low serum 25(OH)D concentrations for several reasons. They stay mostly indoors and so produce little vitamin D from solar ultraviolet-B (UVB), are often obese, and take medications that lower 25(OH)D concentrations [26]. They also are unlikely to take vitamin supplements. Table 3 presents findings regarding serum 25(OH)D concentrations for those with ID or primarily older people living in nursing homes.

**Table 3:** Blood 25(OH)D concentrations among those with ID.

Population	Serum 25(OH)D Concentration	Reference
People with ID in Australia—clinical study	43% had <50 nmol/L	[27]
People with ID in Australia—institution study	57% had <50 nmol/L	[28]
Adults with ID living in nursing homes, Finland	Mean value, 40 nmol/L	[3]
ID patients aged 18–70 years living in Oxfordshire, England	Mean value, 28.8 nmol/L (36.8 nmol/L in summer, 20.3 nmol/L in winter)	[29]

ID, intellectual disability.

### 3.1. Conditions and Diseases That Vitamin D Might Prevent and Treat

#### 3.1.1. Bone Metabolism, Falls and Fractures

Vitamin D was first known as the substance that prevented rickets. Although doctors in the 19th century knew that lack of sunlight was a risk factor for rickets, research did not identify vitamin D as the compound that prevented rickets until the early 20th century [30]. Vitamin D prevents rickets by mediating calcium absorption in the intestines as well as calcium metabolism.

A 2010 study regarding vitamin D deficiency and bone mineralization involved examining bones from vehicular accident victims in Germany. Both blood and bone samples were obtained at autopsy. That study defined *osteomalacia* as a pathologic increase in osteoid volume per bone volume greater than 2%. People with serum 25(OH)D concentration <75 nmol/L met that criterion, but no one with serum 25(OH)D concentrations >75 nmol/L did [31].

Avoiding falls and fractures involves both strong bones and good neuromuscular control. Postural sway is linked to increased risk of falls, and sway was more prevalent in those with serum 25(OH)D concentrations below 30 nmol/L [32]. A study in The Netherlands found that weekly treatment with 8400 IU of vitamin D_3_ reduced postural sway in those with elevated sway at enrollment [33]. A meta-analysis of vitamin D supplements showing reduced risk of falls for elderly people suggested that vitamin D’s effect may be due to improved neuromuscular function with vitamin D supplementation [34]. A pooled analysis of fractures with respect to vitamin D supplementation among those older than 65 years found that “By quartiles of actual intake, reduction in the risk of fracture was shown only at the highest intake concentration (median, 800 IU daily; range, 792 to 2000), with a 30% reduction in the risk of hip fracture (hazard ratio (HR), 0.70; 95% CI, 0.58, 0.86) and a 14% reduction in the risk of any nonvertebral fracture (HR, 0.86; 95% CI, 0.76, 0.96)” [35].

The Endocrine Society recommends serum 25(OH)D concentrations above 75 nmol/L to reduce risk of falls and fractures [36]. People with MCDD often have osteomalacia [37] and osteopenia [38], which has been treated with bisphosphonates [38]. However, a DXA machine, which measures bone mineral density, cannot distinguish low bone mineral density caused by osteoporosis from that caused by osteomalacia. Treating osteomalacia with a bisphosphonate could precipitate severe or even lethal hypocalcemia. In an observational study of patients associated with two U.S. hospitals, favorable response to bisphosphonates was better at higher 25(OH)D concentrations: nonresponder rates were 79% for those with 25(OH)D concentrations <75 nmol/L, 50% for those with 25(OH)D concentrations of 75–100 nmol/L, and only 33% for those with 25(OH)D concentrations >100 nmol/L [39].

To maintain calcium concentration in the blood, the body takes calcium from the intestines or bones according to the balance between parathyroid hormone (PTH) and 1,25-dihydroxyvitamin D [40]. The 25(OH)D concentration–PTH relation has an inverse relation out to at least a serum 25(OH)D concentration of 150 nmol/L [41]. Increased PTH concentrations are associated with increased mortality rates, vascular and valvular calcification, renal failure, heart failure, and cardiovascular disease [40].

However, other factors—such as intake of vitamins C and K as well as calcium and magnesium [42], protein intake [43], and exercise [44,45]—also affect bone strength.

#### 3.1.2. Physical Functioning

Since 25(OH)D concentrations affect muscles and neuromuscular control [32], we can reasonably expect concentrations to also affect physical functioning. A study of two cohorts of elderly people living in Amsterdam examined the relation between serum 25(OH)D concentrations and functional limitations. The study included six functions: walking up and down staircases, dressing and undressing oneself, sitting down and standing up from a chair, cutting one’s toenails, walking outside for 5 minutes without resting, and using one’s own or public transportation. After 3 years of follow-up, those aged at least 65 years at enrollment and with serum 25(OH)D concentrations <75 nmol/L showed more functional limitations (at least two more), whereas those aged 55–65 years at enrollment had more functional limitations after 6 years of follow-up than those with concentrations ≥75 nmol/L [46].

#### 3.1.3. Infectious Diseases

Vitamin D fights bacterial and viral infectious diseases in at least two ways. One is by inducing cathelicidin (also known as LL-37), a polypeptide with antimicrobial and antiendotoxin properties, and defensins [47]. The other is by shifting cytokine production toward diseases less prone to cause inflammation [48,49].

#### 3.1.4. Type A Influenza

The seasonal variation of influenza formed the basis of Cannell’s UVB–vitamin D–influenza hypothesis [50]. Two RCTs supported this hypothesis, one involving black postmenopausal women with a baseline mean 25(OH)D concentration of 48 nmol/L [51], the other involving schoolchildren in Japan [52]. In the black postmenopausal women study, for 312 person-years of taking a placebo with baseline 25(OH)D concentrations 47 ± 21 nmol, 30 colds or influenza cases occurred; for 208 person-years of taking 800 IU/day vitamin D_3_, 8 colds or influenza cases occurred; for 104 person-years of taking 2000 IU/day vitamin D_3_, one cold or influenza case occurred. In the Japan study, supplementation with 1000 IU of vitamin D_3_ per day reduced risk of type A influenza by 67% but did not affect that of type B influenza.

#### 3.1.5. Acute Respiratory Tract Infections, Asthma, and Chronic Obstructive Pulmonary Disease

A June 2013 meta-analysis of the 11 RCTs on vitamin D and RTIs associated vitamin D supplementation with an OR of 0.64 (95% CI, 0.49, 0.84). That analysis also noted that once-daily dosing yielded better effects than bolus dosing (odds ratio = 0.51 *vs.* 0.86; *p* = 0.01) [53].This study supports daily rather than weekly or monthly dosing. Studies have also associated higher 25(OH)D concentrations and vitamin D supplementation with reduced effects of asthma [54,55,56] and chronic obstructive pulmonary disease [57,58]. A meta-analysis of results of vitamin D trials found a statistically significant reduction (relative risk (RR) 0.41, 95% CI, 0.27, 0.63) in asthma exacerbation with vitamin D therapy [59].

#### 3.1.6. Insulin Resistance

In insulin resistance, cells do not respond to insulin, causing high blood sugar. A vitamin D RCT involving insulin-resistant South Asian women living in New Zealand with baseline 25(OH)D concentrations below 50 nmol/L gave half the women 4000 IU of vitamin D_3_ per day and placebos to the other half. Participants reaching serum 25(OH)D concentrations of 80–120 nmol/L showed significantly improved insulin sensitivity [60].

#### 3.1.7. Type 2 Diabetes Mellitus

Mounting evidence indicates that vitamin D reduces risk of developing type 2 diabetes mellitus (T2DM), for which insulin resistance is a risk factor. Several observational studies found that people with higher serum 25(OH)D concentrations had reduced risk of developing T2DM. A meta-analysis of 18 studies found that the RR dropped from 1.0 (95% CI, 0.9, 1.1) at 33 nmol/L to 0.67 (95% CI, 0.45, 0.73) at 100 nmol/L [61]. Some caution regarding this finding is in order because a recent study found that after adjustment for body mass index, the inverse correlation between incidence of T2DM and baseline 25(OH)D concentration was no longer significant [62]. An open-label prospective study in India involving prediabetic individuals with mean age of 48 years followed for a mean of 28 ± 9 months found that having mean baseline 25(OH)D concentration of 95 nmol/L or being supplemented with sufficient vitamin D to raise the final 25(OH)D concentration to 89 nmol/L significantly reduced the conversion to diabetes mellitus compared with baseline or final 25(OH)D concentration of 45 nmol/L [63]. A study in Israel found a beneficial effect of supplementation with 1000 IU of vitamin D per day for patients with T2DM for 12 months in improving the central aortic augmentation index, thereby alleviating some cardiovascular damage [64].

#### 3.1.8. Cardiovascular Disease

Strong evidence from prospective observational studies indicates that serum 25(OH)D concentrations are inversely correlated with cardiovascular disease (CVD). A meta-analysis of 16 studies found that the RR of CVD was 2.2 (95% CI, 1.7, 2.8) for 20 nmol/L, dropping to 1.0 (95% CI, 0.8, 1.2) at 75 nmol/L [65]. The RR for low *vs.* high serum 25(OH)D concentration from these studies was 1.52 (95% CI, 1.30, 1.77). RCTs on those with low 25(OH)D concentrations at enrollment found beneficial effects of vitamin D supplementation. In the Women’s Health Initiative Study, 400 IU of vitamin D_3_ plus 1500 mg of calcium supplementation per day was associated with increased high-density lipoprotein cholesterol and lower low-density lipoprotein cholesterol and triglycerides [66]. The improvements in lipid profiles were most pronounced for those with 25(OH)D concentrations above 100 nmol/L. However, vitamin D RCTs conducted on healthy community-dwelling populations found no beneficial effect of vitamin D supplementation on risk of CVD [67]. Respiratory infections such as influenza can trigger CVD events such as acute myocardial infarction [68], suggesting another way vitamin D might reduce risk of CVD.

#### 3.1.9. Alzheimer’s Disease

Evidence is mounting that vitamin D deficiency is an important risk factor for Alzheimer’s disease (AD). A prospective study in Denmark associated low 25(OH)D concentrations with about a 20% increased risk of both AD and vascular dementia over a 30-year follow-up [69]. Cardiovascular risk factors contribute to risk of AD [70]. As just discussed, vitamin D deficiency is a risk factor for CVD. In early 2014, Gezen-Ak and colleagues reviewed evidence that vitamin D deficiency is an important risk factor for AD. The evidence includes that vitamin D plays roles in protecting the central nervous system, regulating calcium homeostasis, attenuating oxidative stress, and enhancing immune response [71].Another recent paper suggested that vitamin D supplements could be used in therapy for those with AD [72]. A recent cohort study in the U.S. involving 658 elderly people monitored for a mean of 5.6 years found a multivariate-adjusted HR for incident AD of 2.22 (95% CI, 1.02, 4.83) for those with 25(OH)D <25 nmol/L compared with those with 25(OH)D >50 nmol/L [73].

#### 3.1.10. Autism Spectrum Disorder

Evidence increasingly indicates that vitamin D deficiency plays an important role in risk for and progress of autism spectrum disorder. Cannell proposed the vitamin D–autism hypothesis in 2008 [74]. An ecological study of autism prevalence rates for those aged 6–17 years in the United States found significant inverse correlations with solar UVB doses, a proxy for vitamin D production [75]. Researchers in 2014 proposed that a mechanism linking vitamin D deficiency to risk of autism was that vitamin D regulates serotonin synthesis both inside and outside the brain [76]. They also indicated that increasing 25(OH)D concentrations might ameliorate some symptoms of autism. A recent paper reviewed the evidence that vitamin D reduces risk of autism spectrum disorder [77]. Treating those with autism spectrum disorder with vitamin D may reduce symptoms of autism [78,79].

#### 3.1.11. Attention Deficit–Hyperactivity Disorder

People with ID have an increased risk of attention deficit hyperactivity disorder (ADHD) [80]. Two recent papers reported that people with ADHD have lower 25(OH)D concentrations than control subjects [54,81]. Preliminary evidence indicates that increasing 25(OH)D concentrations can reduce symptoms of ADHD [82].

#### 3.1.12. Cancer

Evidence that vitamin D reduces cancer risk comes from several study types. Ecological studies based on geographical variations of solar UVB and cancer incidence or mortality rates furnish good evidence for about 15 cancers [83,84]. Observational studies based on serum 25(OH)D concentrations offer good evidence that vitamin D reduces risk of colorectal and breast cancer [85,86] and aggressive prostate cancer [87]. RCTs offer some evidence that vitamin D reduces risk of cancer [88,89,90], although vitamin D RCTs to date have not been well designed or conducted [13]. As Ref. [13] outlines, vitamin D RCTs should start with an understanding of the 25(OH)D concentration–health outcome relation, measure the 25(OH)D concentration of the prospective participants, include only those whose 25(OH)D concentration is near the lower end of the relation, supplement them with enough vitamin D to raise the concentration to the upper end of the relation, and then remeasure 25(OH)D concentrations. Most vitamin D RCTs to date did not measure baseline 25(OH)D concentrations; those doing so did not reject those with higher 25(OH)D concentrations. Also, until recently, the vitamin D_3_ supplementation was 400 IU/day, although it is rising to 1000–4000 IU/day. Also, for those diagnosed with breast cancer, colon cancer, lung cancer, and lymphoma and who have higher 25(OH)D concentrations, survival rates are much higher [91].

#### 3.1.13. Oral Health

Vitamin D’s role in reducing risk of dental caries has been known since 1928 with a study of vitamin D supplementation in boys in Sheffield, England [92]. The effect was originally thought to be due to better calcium metabolism but has now been linked more strongly to antimicrobial properties through vitamin D’s induction of cathelicidin [93]. Several geographical ecological studies in the mid-20th century inversely correlated solar UVB doses and dental caries [93]. Further evidence comes from controlled trials of vitamin D supplementation and observation of caries incidence. A 2012 review of 24 controlled clinical trials encompassing 2827 participants found a pooled relative-rate estimate of supplemental vitamin D of 0.53 (95% CI, 0.43, 0.65) [94]. Although many of these trials were not modern RCTs, results among them were consistent, giving credibility to the findings. Studies have also linked vitamin D deficiency to periodontal disease [95,96,97]. A recent study in Saudi Arabia found that for older men, “total vitamin D intake ≥ 800 IU was associated with lower odds of severe periodontal disease (OR = 0.67, 95% CI, 0.55, 0.81) and moderate-to-severe ABL (OR = 0.54, 95% CI, 0.30, 0.96) relative to intake <400 IU/day” [98].

#### 3.1.14. Other Health Outcomes

Evidence also indicates that vitamin D reduces risk of cognitive decline [99], hypertension [100], and nonspecific pain [101,102]. These associations are still the subject of ongoing research, but they do offer additional reasons to recommend higher 25(OH)D concentrations for people with MCDD.

#### 3.1.15. All-Cause Mortality Rate

A recent meta-analysis of 32 observational studies found increased HR for 25(OH)D concentrations below 90 nmol/L [103]. The HR for <25 nmol/L was 1.90 (95% CI, 1.63, 2.23), that for 25–48 nmol/L was 1.58 (95% CI, 1.36, 1.84), and that for 50–73 nmol/L was 1.23 (95% CI, 1.06, 1.24). Another meta-analysis found significant RRs for low *vs.* high 25(OH)D concentration in observational studies ranging from 1.14 (95% CI, 1.01, 1.29) to 1.60 (95% CI, 1.32, 1.94), with the exception of secondary prevention cohorts for noncardiovascular, noncancer death, for cancer, cardiovascular, other, and all-cause mortality rates [104].

#### 3.1.16. Health Outcomes in Relation to 25(OH)D Concentrations

Table 4 presents 25(OH)D concentrations above which little additional benefit is found. Several values are based on meta-analyses of 25(OH)D concentration–health outcome relations from observational studies such as those for breast cancer [85], cardiovascular disease [65], T2DM [61], and all-cause mortality rate [103]. Others are based on a variety of studies, including clinical, cohort, and prospective studies, and guidelines by organizations. These results are in line with those reported by Spedding, generally 75–100 nmol/L [105].

**Table 4:** Findings regarding 25(OH)D concentrations related to health conditions from observational studies.

Outcome	Study	Findings with Respect to 25(OH)D	Reference
Athletic performance	Review	100–125 nmol/L	[106]
Bisphosphonate therapy	Clinical study	>100 nmol/L	[39]
Bone quality (poor)	Analysis of people killed in road accidents	75 nmol/L	[31]
Cancer, breast	Meta-analysis	Little change >100 nmol/L	[107]
Cardiovascular disease	Meta-analysis	No change >75 nmol/L	[65]
Dementia	Cohort study	50 nmol/L	[73]
T2DM	Meta-analysis	Little change >75 nmol/L	[61]
Fractures, hip	Prospective study	>63 nmol/L	[108]
Fractures	Prospective study	>75 nmol/L	[109]
Fractures, stress		Reduced <100 nmol/L	[110]
Mortality, all-cause	Meta-analysis	No change >90 nmol/L	[103]
Pain, chronic	Clinical study	>75 nmol/L	[111]
Respiratory infections	Cohort study	>95 nmol/L	[112]

The U.S. Department of Health and Human Services is developing more coordinated and comprehensive approaches to prevent and treat disease in persons with multiple chronic conditions [113]. We hope that these approaches will include increasing 25(OH)D concentrations.

From the relationships between health outcomes and 25(OH)D concentrations, one can estimate the beneficial effects of increasing 25(OH)D concentrations. Table 5 presents findings from several studies, primarily meta-analyses of observational studies. Increasing from 38 to 75 nmol/L reduces average adverse health outcomes by 27%, whereas increasing to 100 nmol/L reduces outcomes by 36%.

**Table 5:** Estimated reductions in disease rates by increasing 25(OH)D concentrations.

Outcome	75 *vs.* 38 nmol/L	100 *vs.* 38 nmol/L	Reference
Cancer, breast	0.59	0.48	[107]
Cardiovascular disease	0.71	0.71	[65]
T2DM	0.76	0.62	[61]
Fractures, nonvertebral	0.81		[114]
Mortality, all-cause	0.72	0.64	[103]
Periodontal disease	0.67		[115]
Respiratory infections, upper respiratory	0.85	0.76	[116]
Mean values	0.73	0.64	
Mean values for those with data for 75 and 100 nmol/L	0.73	0.64	

A few reports found adverse health effects for higher 25(OH)D concentrations, the most important being hypercalcemia, which generally does not occur for 25(OH)D concentrations below 500 nmol/L [117]. Achieving this concentration is highly unlikely unless someone takes more than 50,000 IU of vitamin D daily for a prolonged period or mistakenly overdoses with high-concentration vitamin D supplements with 1million IU of vitamin D [118]. We discuss findings of J- or U-shaped 25(OH)D concentration–health outcomes later.

### 3.2. Reviews of Vitamin D Benefits, Requirements, Recommendations

Several health organizations and vitamin D working groups have reviewed the evidence of health benefits of vitamin D and recommended desirable serum 25(OH)D concentrations and vitamin D_3_ supplementation. Many molecular mechanisms of vitamin D’s action are well known [119]. Table 6 summarizes recommendations for those likely to be vitamin D deficient. The general consensus of these recommendations is that serum 25(OH)D concentrations should be at least 75 nmol/L and up to 125 nmol/L and that reaching these concentrations takes about 1000–2000 IU of vitamin D_3_ per day. A recent paper also analyzed optimal concentration on the basis of three diverse findings (zero correlation between 25(OH)D and PTH above a threshold, support of lactation, and ancestral values), concluding that 100–130 nmol/L was optimal and could be achieved with all-source inputs of 4000–6000 IU per day [13]. The task force considered input from these organizations and working groups in making recommendations for people with MCDD.

**Table 6:** Vitamin D recommendations by organizations and groups.

Organization	Intended Group	Serum 25(OH)D Concentration (nmol/L)	Vitamin D_3_ (IU/day)	Vitamin D_3_ UL (IU/day)	Reference
Vitamin D experts	Elderly and institutionalized individuals	75–100	800		[120]
Endocrine Society	Patients at risk of vitamin D deficiency, 1–18 years	75	600–1000	4000	[36]
	Patients at risk of vitamin D deficiency, ≥19 years	75	1500–2000	4000	[121]
European Menopause and Andropause Society	Women with vitamin D deficiency related to osteoporosis	>75	800–1200		[25]
French Group of Geriatrics and Nutrition	Elderly nursing home residents	75–100	1000		[122]
Central European Guidelines	Obese children and adolescents	75–125	1200–2000		[123]
	Obese adults and the elderly	75–125	1600–4000		[123]
ESCEO	Adults	>50	800–1000		[124]
	Elderly at risk	>75			[124]
American Geriatrics Society	Adults ≥70 years	>75	4000		[125]

ESCEO, European Society for Clinical and Economic Aspects of Osteoporosis and Osteoarthritis; UL, upper limit.

#### 3.2.1. Institute of Medicine Report

The Institute of Medicine (IOM) issued a report in 2010 on dietary requirements for calcium and vitamin D for people living in North America. Its recommendations for vitamin D intake to achieve a serum 25(OH)D concentration of 50 nmol/L were 400 IU/day for infants younger than 1 year, 600 IU/day for those aged 1–70 years, and 800 IU/day for those aged ≥71 years [126]. The abstract of that paper stated, “The Committee concluded that available scientific evidence supports a key role of calcium and vitamin D in skeletal health, consistent with a cause-and-effect relationship and providing a sound basis for determination of intake requirements. For extraskeletal outcomes, including cancer, cardiovascular disease, diabetes, and autoimmune disorders, the evidence was inconsistent, inconclusive as to causality, and insufficient to inform nutritional requirements. Randomized clinical trial evidence for extraskeletal outcomes was limited and generally uninformative”. The vitamin D research community severely criticized the IOM report. For example, “The IOM recommendations for vitamin D fail in a major way on logic, on science, and on effective public health guidance. Moreover, by failing to use a physiological referent, the IOM approach constitutes precisely the wrong model for development of nutritional policy” [127]. The only evidence that the IOM committee found acceptable as a basis for policy recommendations was from RCTs and observational studies regarding bone health. Among other things, IOM misinterpreted the observational study it used to set the 25(OH)D concentration to 50 nmol/L; the authors of the study concluded from the data that 75 nmol/L was the appropriate concentration [31].

The levels of evidence for evidence-based medicine place systematic reviews of RCTs at the top but permit observational studies and mechanism-based reasoning in the absence of RCTs [128]. The recommendations in Table 6 were based largely on observational studies and mechanisms. As noted, vitamin D RCTs have in general been poorly designed. A recent paper argued that absence of supportive vitamin D RCTs should not undermine the observational studies finding beneficial effects [129]. The IOM report also noted that “Guidelines regarding the use of serum markers of vitamin D status for medical management of individual patients and for screening were beyond the scope of the Committee’s charge, and evidence-based consensus guidelines are not available. However, these issues should be addressed by appropriate federal agencies and professional organizations in light of the findings in this report.” Most of the recommendations in Table 6 were done by professional organizations for the benefit of those they serve. For example, one stated, “The objective was to provide guidelines to clinicians for the evaluation, treatment, and prevention of vitamin D deficiency with an emphasis on the care of patients who are at risk for deficiency” [36].

Finally, since the IOM report was published (29 November, 2010), 13,535 publications with vitamin D in the title or abstract have been published at PubMed.gov as of 23 February, 2015, compared with 27,775 published before that date. This paper cites many of these publications. Until about 2000, most papers published on the use of vitamin D in clinical trials was related to musculoskeletal effects; now, however, the evidence of benefits for nonskeletal effects is still accruing. The IOM listed 4000 IU/day vitamin D as the upper limit of supplementation. However, they noted that no adverse effects had been reported for less than 10,000 IU/day (see [130]). A recent paper studied the dose–response relation for 25(OH)D and serum calcium as a function of vitamin D supplementation. For daily doses of 10,000 IU/day, the mean 25(OH)D concentration was above 150 nmol/L (155 nmol/L) only for the underweight group; for the obese group, the mean concentration was 110 nmol/L [131]. Serum calcium was nearly unchanged for up to 20,000 IU/day.

#### 3.2.2. U- and J-Shaped 25(OH)D Concentration–Health Outcome Relations

Several observational studies reported a J- or U-shaped 25(OH)D concentration–health outcome relation with the following factors: all-cause mortality [132,133]; adverse cardiac and cerebrovascular events in cardiac surgery [134]; frailty [135]; hospital mortality [136]; and immunoglobulin E [137]. The mortality rate findings are not supported in a meta-analysis of 32 studies [103]. Such studies have been cited to warn against supplementing with too much vitamin D or raising 25(OH)D concentrations too high. However, most of these studies are on elderly people and the highest 25(OH)D quintile was generally above 100 nmol/L, a value generally reached in the U.S. and Europe through vitamin D supplementation. None of those studies seems to have asked participants when they started taking supplements. For frailty, although a U-shaped relation emerged for older women, a nearly linear inverse 25(OH)D concentration-frailty relation was present for men [138]. The difference between men and women is consistent with the fact that women are often advised to start taking vitamin D supplements after menopause, and that taking vitamin D late in life cannot overcome all the adverse effects of low 25(OH)D concentrations earlier in life. No mechanisms have been proposed to explain most of the J- or U-shaped relations. 

A recent paper reporting an observational study of inflammation with respect to 25(OH)D concentration stated, “On the other hand, the U-shaped association may be an artifact, determined by the small proportion of subjects with 25(OH)D in the target range (25(OH)D ≥30 ng/mL: 13.1% of subjects; 25(OH)D ≥40ng/mL; 3.0% of subjects). The majority of our study population (76.3%) had 25(OH)D concentrations <25 ng/mL, a range in which hs-CRP decreased with increasing 25(OH)D. Additionally, we cannot exclude that subjects with high 25(OH)D concentrations had not acknowledged taking vitamin D supplements in the SHIP examination, which might have biased the analyses. Overall, there is no final explanation for the U-shaped association between 25(OH)D and hs-CRP in our study population and we suggest assessing it in future studies” [139].

Another recent paper examined whether 25(OH)D concentrations >100 nmol/L reduced 1,25-dihydroxyvitamin D concentrations to account for the U-shaped relation for postoperative recovery after cardiac surgery; the answer was no [140]. However, that paper noted that impaired kidney function as measured by estimated glomerular filtration rate is associated with lower conversion of 25(OH)D to 1,25(OH)_2_D, which could account for some adverse effects associated with higher 25(OH)D concentrations. Reports that seem most credible—both for 25(OH)D >125 nmol/L—revealed increased immunoglobulin E, a marker of allergic responses [137], as well as reduced cognitive performance [141]. Men had increased risk of hypogonadism for 25(OH)D concentrations >100 nmol/L [142]. However, these reports need further confirmation.

#### 3.2.3. Reports of Adverse Events Associated with High dose Vitamin D

One of the dangers of vitamin D supplementation is risk of hypercalcemia or blood calcium levels that are too high. Vitamin D intoxication can result in an elevated serum calcium and serum phosphorus level. Constitutional symptoms include confusion, nausea, constipation, polyuria and polydipsia, decreased heart rate and arrhythmias [143]. The long-term consequences include soft tissue calcification of the blood vessels, nephrocalcinosis and kidney stones. Generally, hypercalcemia does not occur for vitamin D supplementation less than 40,000 IU/day [144]. However, sometimes high 25(OH)D concentrations are reached by accident, such as a manufacturing and labeling errors [118,143,145]. “Hydration, diuretics and prednisone induced a progressive reduction of calcium levels” [143]. It can take several months to a year for 25(OH)D concentrations to return to normal, although hypercalcemia disappeared below 25(OH)D concentrations of 1000 nmol/L in one study [118].

#### 3.2.4. Reverse Causality

Concern has been raised that 25(OH)D concentration–health outcome relations found in observational studies could be due to reverse causation, that is, that having disease affects 25(OH)D concentrations. This concern has been raised in large part since vitamin D RCTs generally have not confirmed the findings of observational studies [146]. This effect is most likely to be found in cross-sectional studies of disease prevalence, and most authors are careful to acknowledge this possibility. However, reverse causation is not thought to affect prospective studies with long follow-up times, especially if health outcomes occurring in the first year or two are omitted. Those outcomes might be due to undiagnosed disease, since it is assumed that the health outcome developed after measurement of 25(OH)D concentration. However, reverse causation might affect case–control studies in which 25(OH)D concentrations are measured near time of diagnosis.

Breast cancer is one health outcome for which reverse causality is often claimed for case-control studies since nested case-control studies do not find significant inverse correlations with 25(OH)D concentrations for follow-up times longer than 3 years [86]. A recent paper argues that case-control studies do not show evidence of reverse causality for breast cancer since the 25(OH)D concentration-incidence relations for 10 studies overlay each other very well [107]. One of the 10 studies included in the meta-analysis used 25(OH)D concentrations measured about 1year before diagnosis and had similar findings to the other studies, which measured 25(OH)D concentrations shortly after diagnosis. The reason for the disparity between case-control studies and nested case-control studies is attributed to the rapid development of breast cancer tumors. Breast cancer screening is recommended annually, whereas colorectal cancer screening is recommended every 10 years.

#### 3.2.5. Randomized Controlled Trials

Several recent papers have pointed out that vitamin D RCTs do not support the findings of observational studies [67,146,147]. In response to the paper by Autier [146], three of us analyzed all the RCTs examining the effect of vitamin D on biomarkers of inflammation. Half of the trials with baseline 25(OH)D concentration <48 nmol/L resulted in significant inverse correlations between vitamin D supplementation and inflammation, whereas only 25% of those with higher baseline 25(OH)D concentrations did [148]. One problem with most vitamin D RCTs conducted to date is that they have largely been based on guidelines for pharmaceutical drugs, which assume that the trial is the only source of the agent and that a linear dose–response relation is in effect. Vitamin D satisfies neither assumption since UVB exposure, diet, and supplements are common sources of vitamin D. Another problem is that those conducting the trials generally did not design the trials to evaluate the 25(OH)D concentration-health outcome relation. In a recent paper, Heaney outlined guidelines for trials of nutrients such as vitamin D.

The important steps include starting with an understanding of the 25(OH)D concentration-health outcome relation, measuring 25(OH)D concentrations of potential participants, enrolling only those with 25(OH)D concentrations near the low end of the relation, supplementing them with enough vitamin D to raise 25(OH)D concentrations to near the upper end of quasi-linear region of the relation, remeasuring 25(OH)D concentrations, and ensuring that important cofactors have been optimized [13]. Very few vitamin D RCTs conducted to date satisfy these guidelines; thus, few found significant effects. Also, some question exists of whether the trials were conducted at the right age and for a long enough period.

#### 3.2.6. Vitamin D_3_ (Cholecalciferol) *vs.* Vitamin D_2_ (Ergocalciferol)

Vitamin D_3_ (cholecalciferol) is synthesized in human skin, whereas vitamin D_2_ (ergocalciferol) comes from yeast and fungi. Most vitamin D supplements are vitamin D_3_. Reports of the effectiveness of the two types conflict. In a study supplementing healthy adults with 50,000 IU of vitamin D_2_ or D_3_, vitamin D_3_ was 87% more potent in raising and maintaining 25(OH)D concentrations [149].

A 2012 study with 50,000 IU of vitamin D_2_ supplementation every other week increased total serum 25(OH)D concentration from 78 to 120 nmol/L but lowered 25(OH)D_3_ concentration from 68 to 35 nmol/L [150]. The most recent study found that treating those with T2DM with 50,000 IU of vitamin D_2_ per day for 10 days yielded increases comparable to those taking 40,000 IU of vitamin D_3_ daily for 10 days [151]. Heavier people require larger vitamin D doses [152]. A meta-analysis of all-cause mortality rate from vitamin D supplementation trials found a RR of 0.89 (95% CI, 0.80, 0.99) for trials using vitamin D_3_ and 1.04 (95% CI, 0.97, 1.11) for trials using vitamin D_2_ [104]. In other words, vitamin D_3_ significantly reduced risk of death, whereas vitamin D_2_ did not.

Another consideration is that 50,000-IU vitamin D_2_ capsules can be prescribed, but 50,000-IU vitamin D_3_ capsules cannot. However, prescription-grade vitamin D_3_ is available at lower cost than vitamin D_2_ (Bio-Tech Pharmacal, Fayetteville, AR, USA). Vitamin D capsules of 50,000 IU can be given once per month, a daily average of 1640 IU. Alternatively, for example, 20,000 IU per week could be given, a daily average of 2860 IU. Since 25(OH)D has a half-life in the blood of 4–6 weeks, such dosing is acceptable.

#### 3.2.7. Diet and 25(OH)D Concentrations

Few foods contain vitamin D. The primary food sources of vitamin D in the United States are fatty fish and vitamin D-fortified milk or other foods; however, many other countries do not fortify any food with vitamin D. In the United States, the mean daily vitamin D intake from food for adults is about 250 IU [153]. However, some diets provide more vitamin D than others. A UK study found that meat eaters had 25(OH)D concentrations 20 nmol/L higher than those of vegans [154]. Fish eaters had slightly lower concentrations than those of meat eaters, whereas vegetarians, who may eat milk and eggs, had concentrations about halfway between those of meat eaters and vegans. Meat evidently has vitamin D as 25(OH)D, which tests generally do not measure. Diet has important effects on health, but the amount of vitamin D derived from food is not enough to raise 25(OH)D concentrations to recommended values.

#### 3.2.8. Testing Serum 25(OH)D Concentrations

Since the recommendations are primarily for serum 25(OH)D concentrations, those with MCDD should have their serum 25(OH)D concentrations tested before beginning supplementation as well as after 6 months to see whether the dose is correct, then annually thereafter. Achieved 25(OH)D concentrations vary considerably with respect to oral vitamin D intake [155]. Taking vitamin D_2_ and vitamin D_3_ also yields different 25(OH)D concentrations. Apparently, some individuals can take up to 1 year to obtain a steady-state serum level of 25(OH)D, so a 3- or 6-month level is not necessarily the maximum obtainable.

However, 25(OH)D assays still have some problems. Several approaches for measuring 25(OH)D concentrations exist, including immunoassays, high-performance liquid chromatography, and liquid chromatography–tandem mass spectrometry. Liquid blood or dried blood spots can also be used. Recent reports compared automated immunoassays with liquid chromatography-tandem mass spectrometry methods [156,157]. Both methods have generally good reproducibility and low bias. Other assays did not compare well. Thus, investigating the assay used to measure 25(OH)D concentration is important.

The international Vitamin D External Quality Assessment Scheme sends blood samples to laboratories throughout the world to check measurement accuracies. By 2011, intra-laboratory imprecision was down to 15% [158]. From a clinical point of view, a good policy would probably be to ask the assay company for accuracy and repeatability values of 25(OH)D measurements and whether vitamin D_2_ and vitamin D_3_ are measured separately or together.

## 4. Conclusions

This review summarizes evidence that vitamin D has important health benefits for those with MCDD as well as others. The vitamin D recommendations by health organizations and vitamin D researchers are that 25(OH)D concentrations for optimal health are in the range of 75 to 100 or 125 nmol/L (30 to 50 ng/mL) and that to reach these concentrations takes 800 to 4000 IU/day vitamin D_3_. However, since solar UVB exposure is the natural way to obtain vitamin D_3_, and since there appear to be additional health benefits associated with solar UV exposure, sensible solar UVB exposure should also be considered when the sun is high enough that one's shadow is shorter than one’s height. We hope that physicians who treat those with MCDD will incorporate vitamin D supplementation in their practice. Tracking results of vitamin D supplementation, either formally or informally, would also be advisable.
